# The surgical efficiency of Kirschner wire sleeve-assisted removal of elastic intramedullary nails: a comparative study

**DOI:** 10.3389/fped.2025.1689452

**Published:** 2026-01-29

**Authors:** Xue-Tang Lin, Lin-Xiong Wang, Xiao-Cong Chen, Wei-Peng Gong, Shang-Guan Shang-Lin, Dong-Qing Huang, Shu-Mu Yang, Na-Ling Yi, Long-Feng Tang

**Affiliations:** 1 Department of Orthopedics, Anxi County Hospital, Anxi, Fujian, China; 2 Chronic Disease Management Center, Anxi County Hospital, Quanzhou, Fujian, China

**Keywords:** elastic intramedullary nail, fracture fixation, implant extraction, K-wire sleeve, minimally invasive surgery, operative efficiency, pediatric orthopedics

## Abstract

**Background:**

Elastic intramedullary nails (ESIN) are widely used for pediatric fractures; however, their removal poses technical challenges. Currently, there are limited reports on improvements in ESIN removal techniques. This study aimed to explore the clinical efficacy of Kirschner Wire (K-wire) sleeve-assisted ESIN removal surgery and to provide new references for ESIN extraction in orthopedic surgery.

**Methods:**

This retrospective study included 32 patients who underwent ESIN removal surgery at our hospital between October 2020 and July 2024. Patients were retrospectively assigned to two groups based on surgical method: the conventional instrument removal group and the K-wire cannula-assisted removal group. The efficacy of K-wire sleeve-assisted ESIN removal surgery was then compared with that of the traditional method.

**Results:**

The K-wire sleeve group (observation group, *n* = 17) exhibited a significantly shorter operative time (4.65 ± 1.12 vs. 11.33 ± 1.47 min/nail, *p* < 0.001) and significantly smaller incisions (0.95 ± 0.11 vs. 1.43 ± 0.33 cm/nail, *p* < 0.001) when compared to traditional methodology (control group, *n* = 15). Among the 32 patients, no cases of postoperative incision infection, intraoperative nerve injury, or vascular injury were observed.

**Conclusion:**

The Kirschner Wire (K-wire) sleeve-assisted ESIN removal technique provided a minimally invasive and cost-effective alternative to traditional methods.

## 1 Introduction

Elastic intramedullary nails (ESIN) are widely utilized for pediatric fracture fixation due to their minimally invasive nature, excellent clinical outcomes, and preservation of periosteal blood supply. Recognized for their unparalleled efficacy, ESIN remain as the gold-standard treatment for pediatric long bone fractures (1). The application of ESIN has also been extended to adult fractures involving the clavicle, fibula, and other anatomical sites (2). The timely removal of ESIN post-healing was considered essential to mitigate complications associated with prolonged retention, including infection risk, implant migration or fracture, and activity-related localized pain (3).

**Figure 1:**
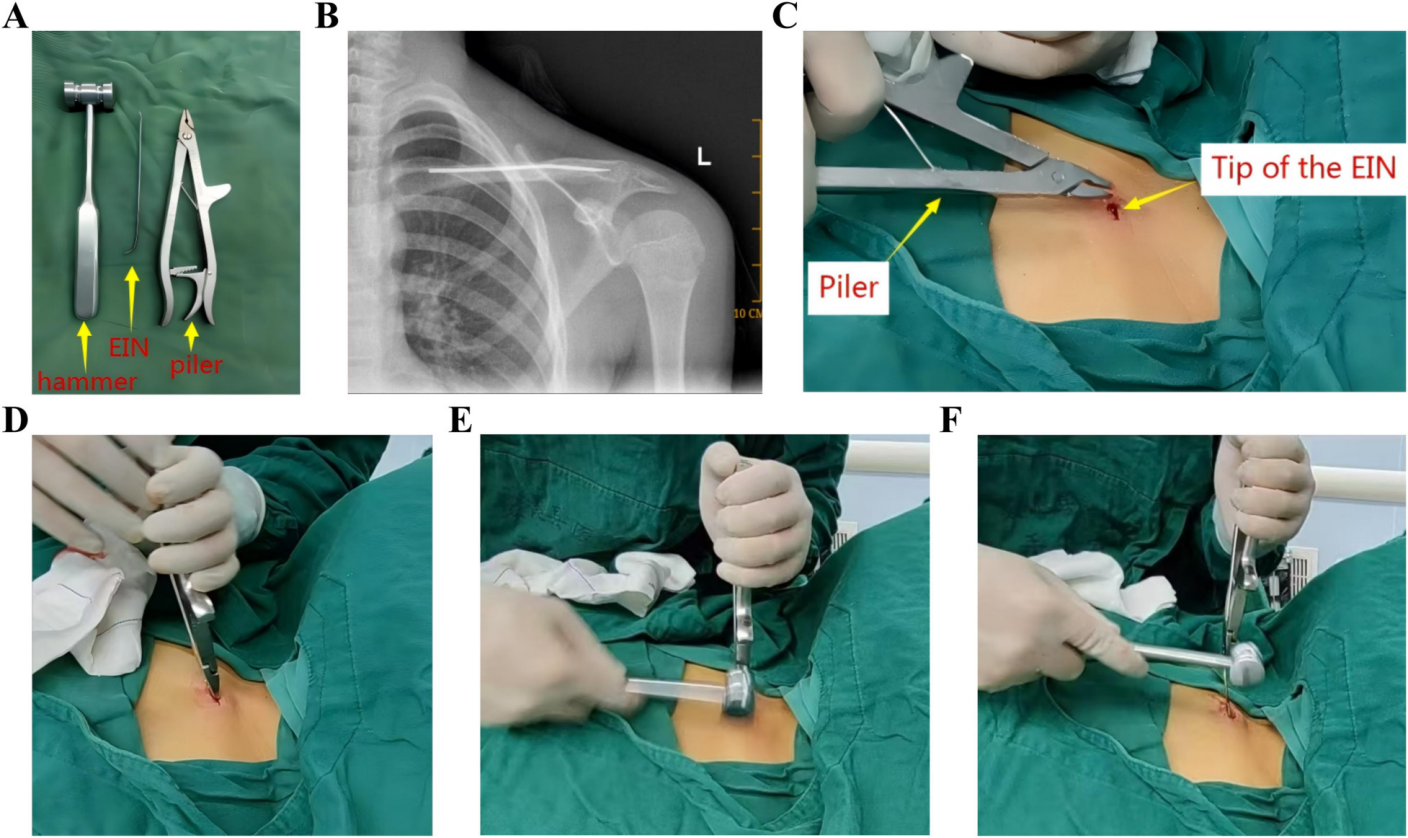
Conventional ESIN extraction steps. **(A–F)** Direct clamping and mallet-assisted withdrawal; **(C)** design limitations of standard extraction forceps: Thick, blunt-ended jaw tips. ESIN, Elastic intramedullary nails.

**Figure 2:**
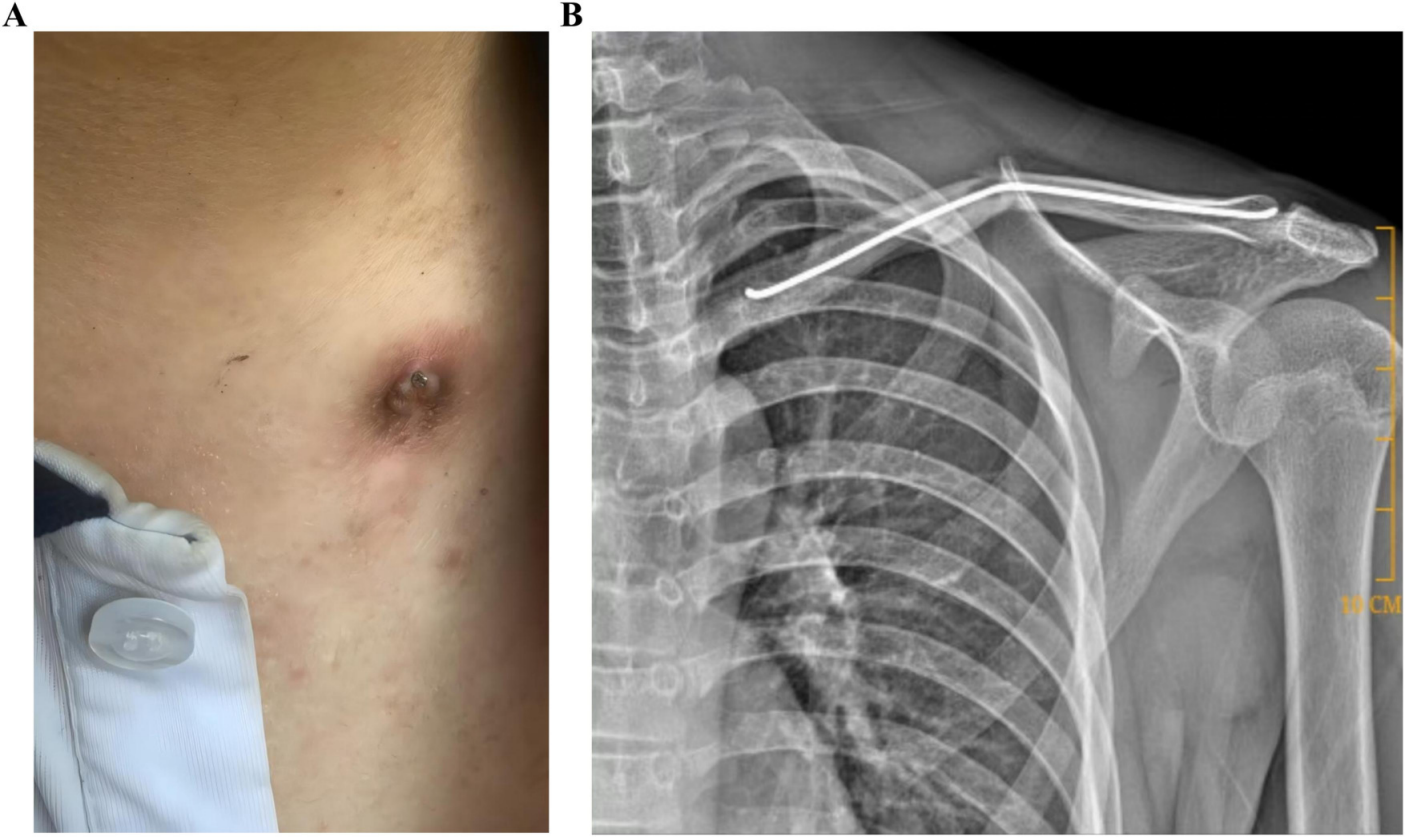
Complications of excessive ESIN tip angulation. **(A,B)** Soft tissue irritation. ESIN, elastic intramedullary nails.

**Figure 3:**
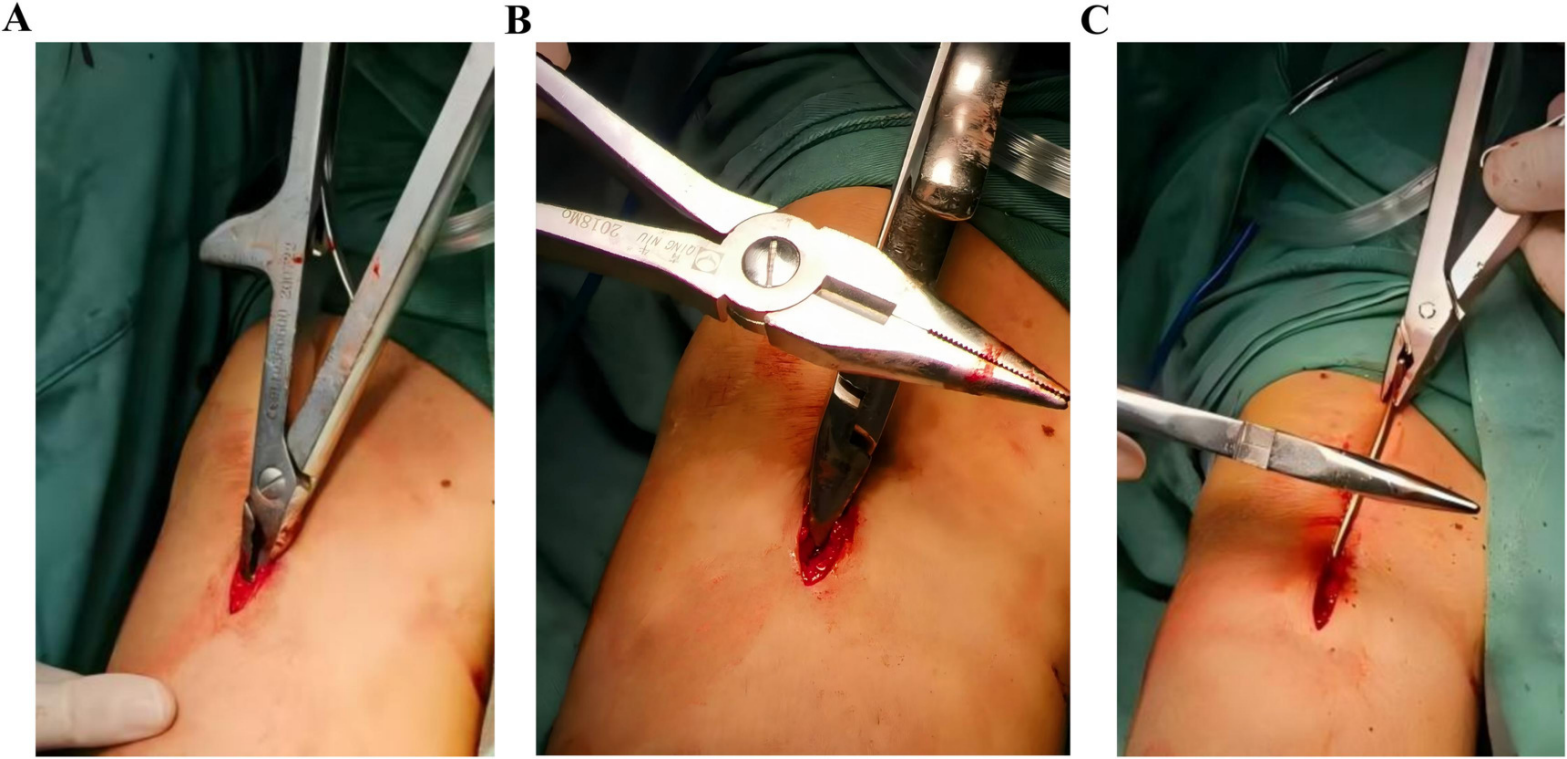
Instrument-based grasping of the needle tail. **(A–C)** Incision extension requirements.

However, current ESIN extraction techniques faced three critical challenges. Firstly, instrument limitations; the traditional extraction method involved directly clamping the tip of an ESIN with extraction forceps and pulling it out (Figures 1A–F). However, the thickened tips of standard ESIN removal forceps (Figure 1A) made it difficult to securely grasp the nail tip. Secondly, nail tip angulation; if the angle of bend for the ESIN tip protruding outside the bone was excessive, this may irritate the soft tissues and skin, leading to pain, bursitis, infection with cutaneous exposure, and even restricted joint mobility (Figures 2A,B). Thirdly, the length of the nail tip. To allow removal instruments to clamp the nail tip under direct visualization, the tip needed to be fully exposed, which often required enlargement of the original incision (Figures 3A–C). In cases where the ESIN tip was positioned close to the cortical bone with only a short exposed segment, direct clamping frequently failed to maintain secure fixation and resulted in slippage. Some cases even required chiseling of the cortical bone to adequately expose and grasp the nail tip.

**Figure 4:**
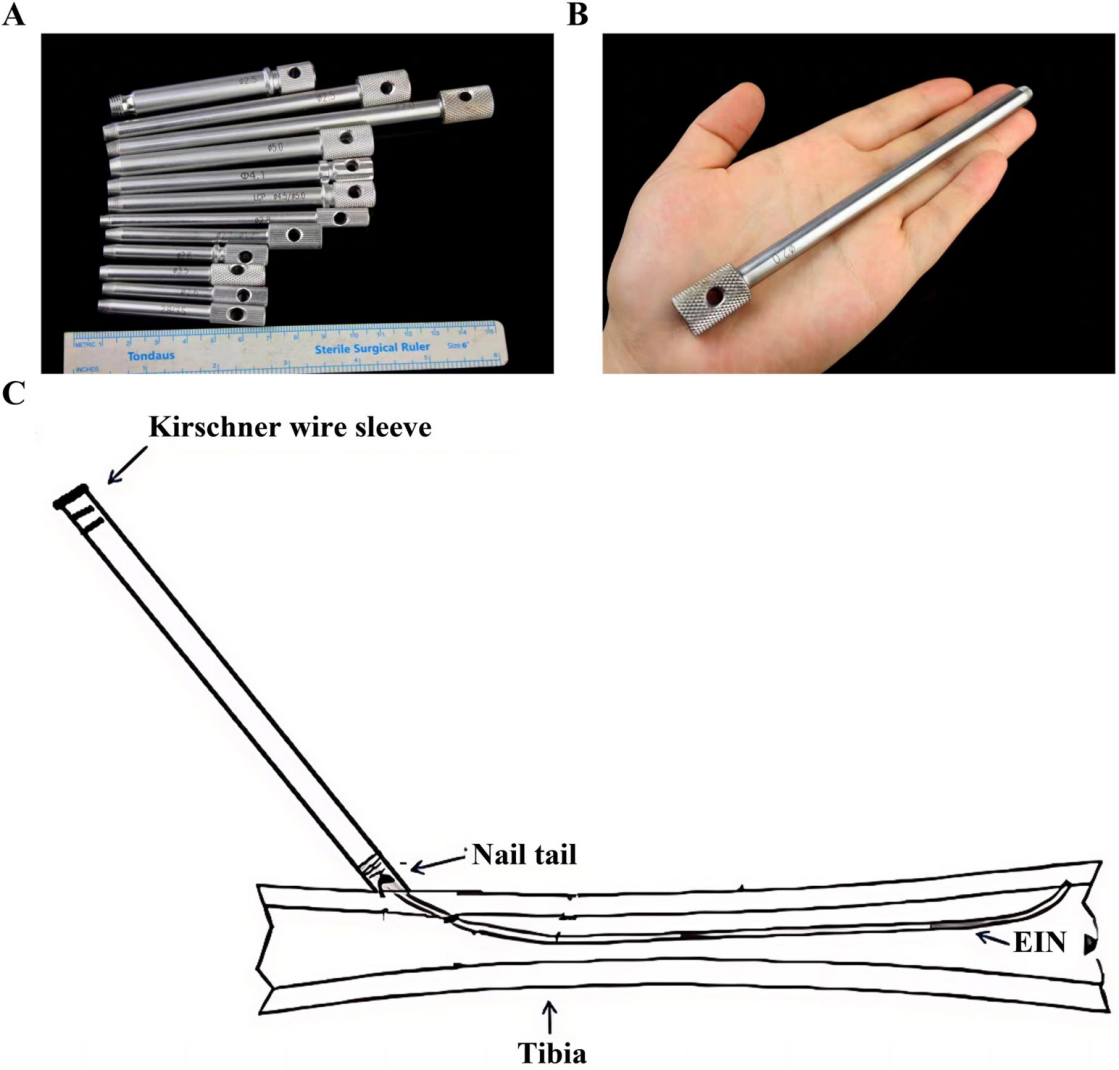
K-wire sleeve specifications. **(A,B)** Diameter spectrum (1.5–5.0 mm) and compatibility with ESINs; **(C)** insertion and controlled bending mechanism; K-wire, Kirschner Wire.

To establish an instrument combining both bending-resistant structural integrity and routine operating room availability for ESIN extraction, we utilized Kirschner Wire (K-wire) sleeves, commonly employed in orthopedic procedures (Figures 4A–C), as auxiliary devices. This study aimed to evaluate the advantages of K-wire cannula-assisted ESIN removal surgery over traditional methods in terms of surgical efficiency and complication rates, thereby providing a safer, more effective, and economically viable technical solution for clinical ESIN extraction.

## 2 Materials and methods

### 2.1 Clinical data

This study was a retrospective analysis involving 32 patients who underwent ESIN removal at our hospital between October 2020 and July 2024. Patients were randomly assigned to groups using a computer-generated sequence with a block randomization. Patients were included if they had a fracture that had completely healed with no surgical contraindications. Patients were excluded if there was evidence of fracture non-union or the presence of significant surgical contraindications. The patients were retrospectively assigned to two groups based on surgical method: a conventional instrument removal group (control group, *n* = 15) and a K-wire sleeve-assisted removal group (observation group, *n* = 17).

The study was approved by the Ethics Committee of Anxi County Hospital, and informed consent was obtained from all patients. All research methods complied with the Declaration of Helsinki. All images presented herein are derived from surgical cases at our institution and were obtained with the patient consent.

### 2.2 Surgical methods

All procedures in both groups were performed by the same surgical team.

#### 2.2.1 Control group: ESIN removal with conventional instruments

**Figure 5:**
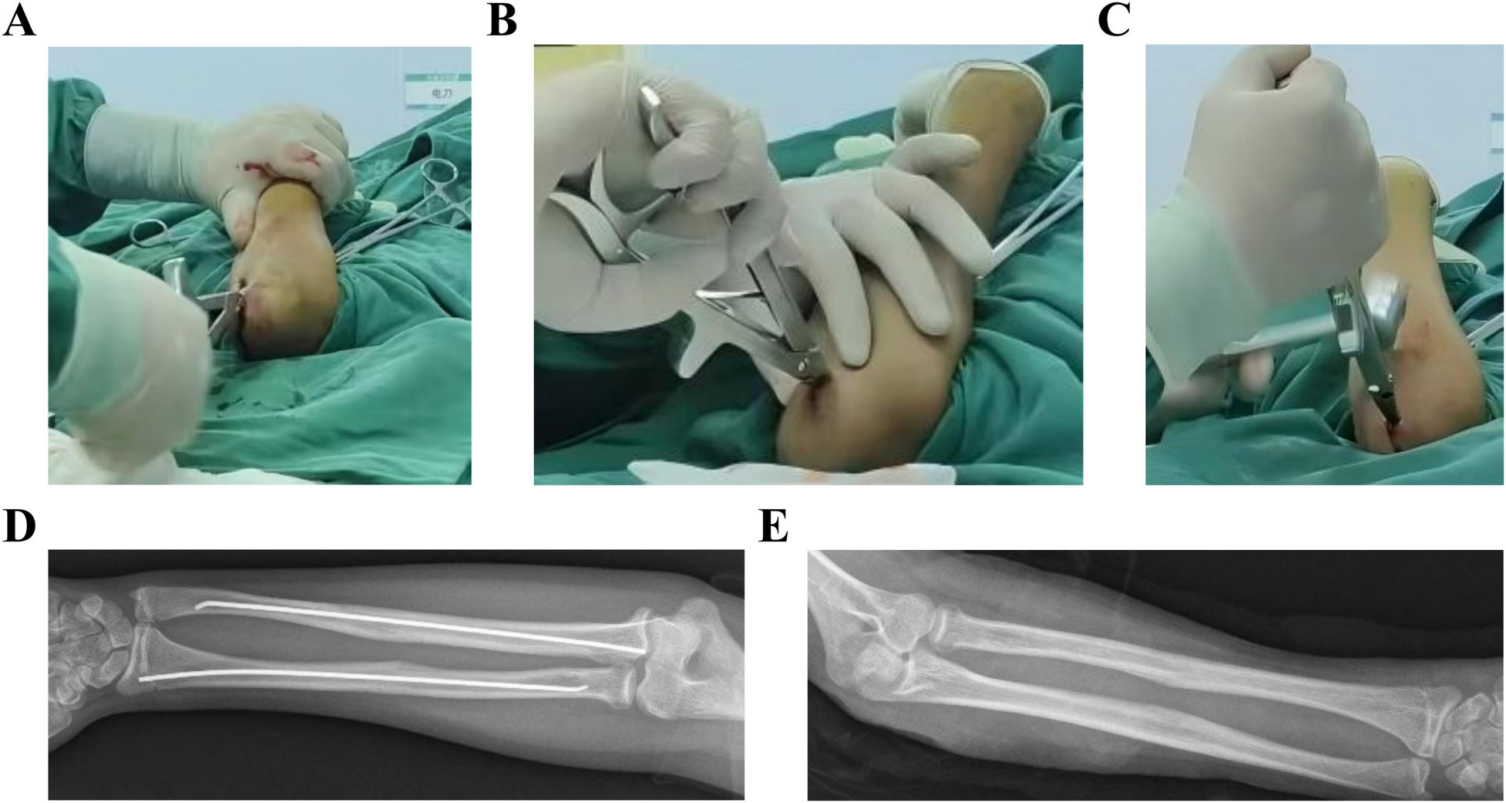
Conventional ESIN removal. **(A–E)** Incision enlargement and mallet-assisted extraction. ESIN, elastic intramedullary nails.

The original incision size was used for all patients. Layered dissection was performed to expose the ESIN tip; if exposure was challenging, the incision was appropriately enlarged. The ESIN tip was grasped with clamping instruments and slightly bent. The clamp was then struck with a hammer to extract the ESIN. The wound was irrigated, closed in layers with sutures, and covered with sterile dressings to complete the procedure. Postoperative wound dressings were changed regularly (Figures 5A–E).

#### 2.2.2 Observation group: K-wire sleeve-assisted ESIN removal

**Figure 6:**
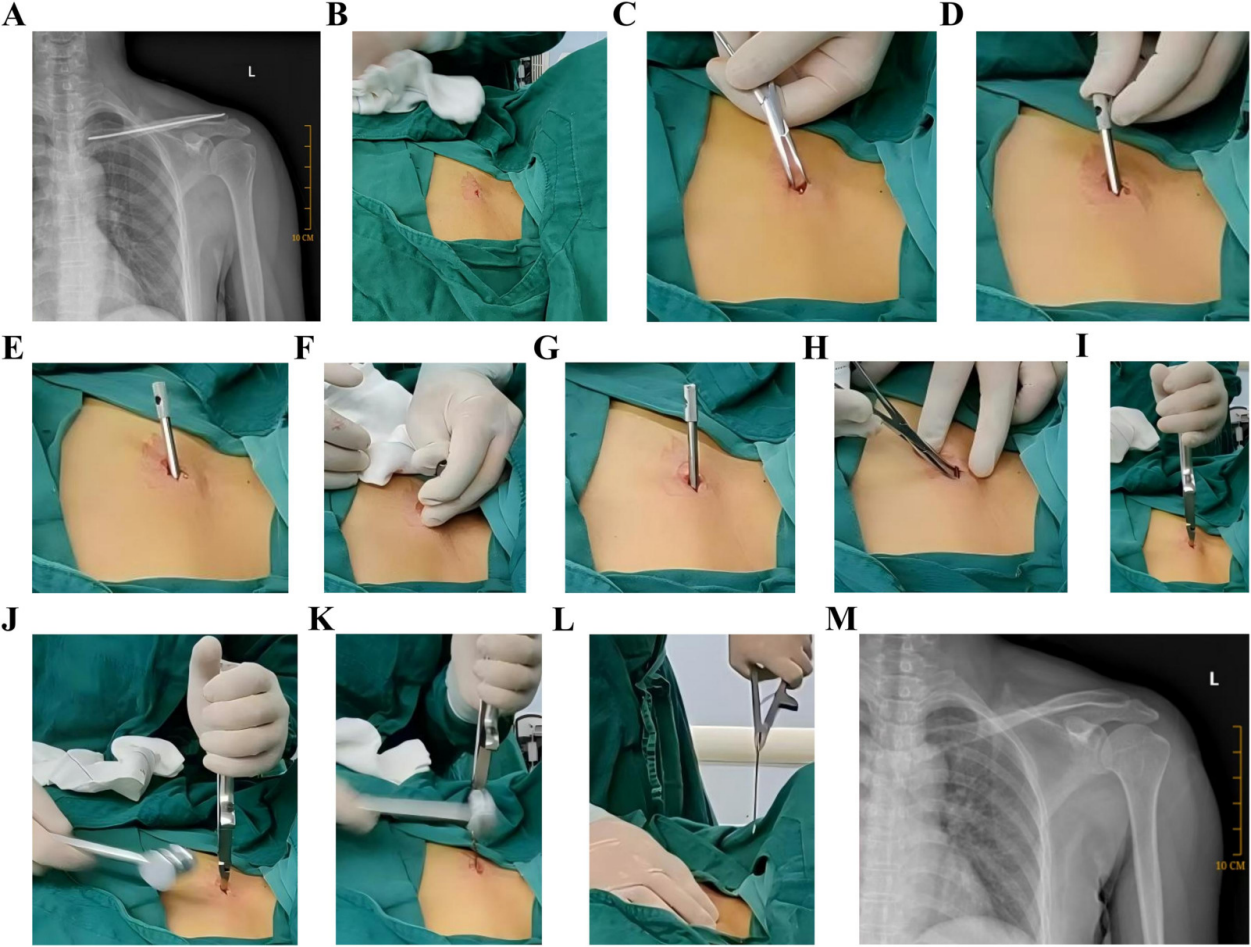
K-wire sleeve-assisted ESIN removal from an adult clavicle. **(A)** Postoperative radiograph confirming fracture union (1-year follow-up); **(B)** original incision reopening to expose ESIN terminus; **(C)** hemostat-assisted exposure expansion; **(D)** Sleeve insertion along the ESIN terminus without incision enlargement; **(E)** initial angulation (30°) of the ESIN terminus; **(F)** controlled bending via the sleeve; **(G,H)**. Final angulation (70–90°); near-orthogonal configuration); **(I)** perpendicular engagement of clamping instruments; **(J–M)** mallet-assisted ESIN extraction. ESIN, elastic intramedullary nails; K-wire, Kirschner Wire.

**Figure 7:**
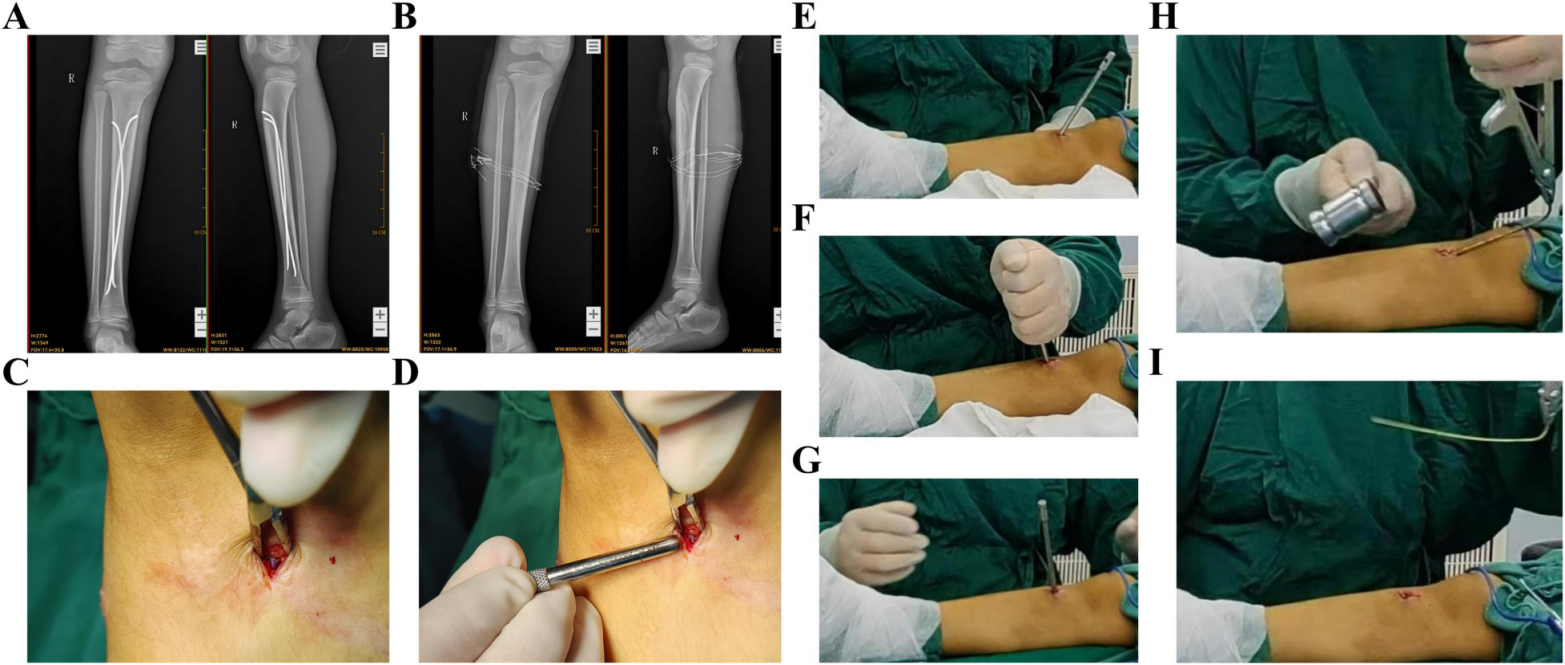
K-wire sleeve-assisted tibial ESIN removal. **(A–I)** Stepwise procedural demonstration. ESIN, elastic intramedullary nails; K-wire, Kirschner Wire.

**Figure 8:**
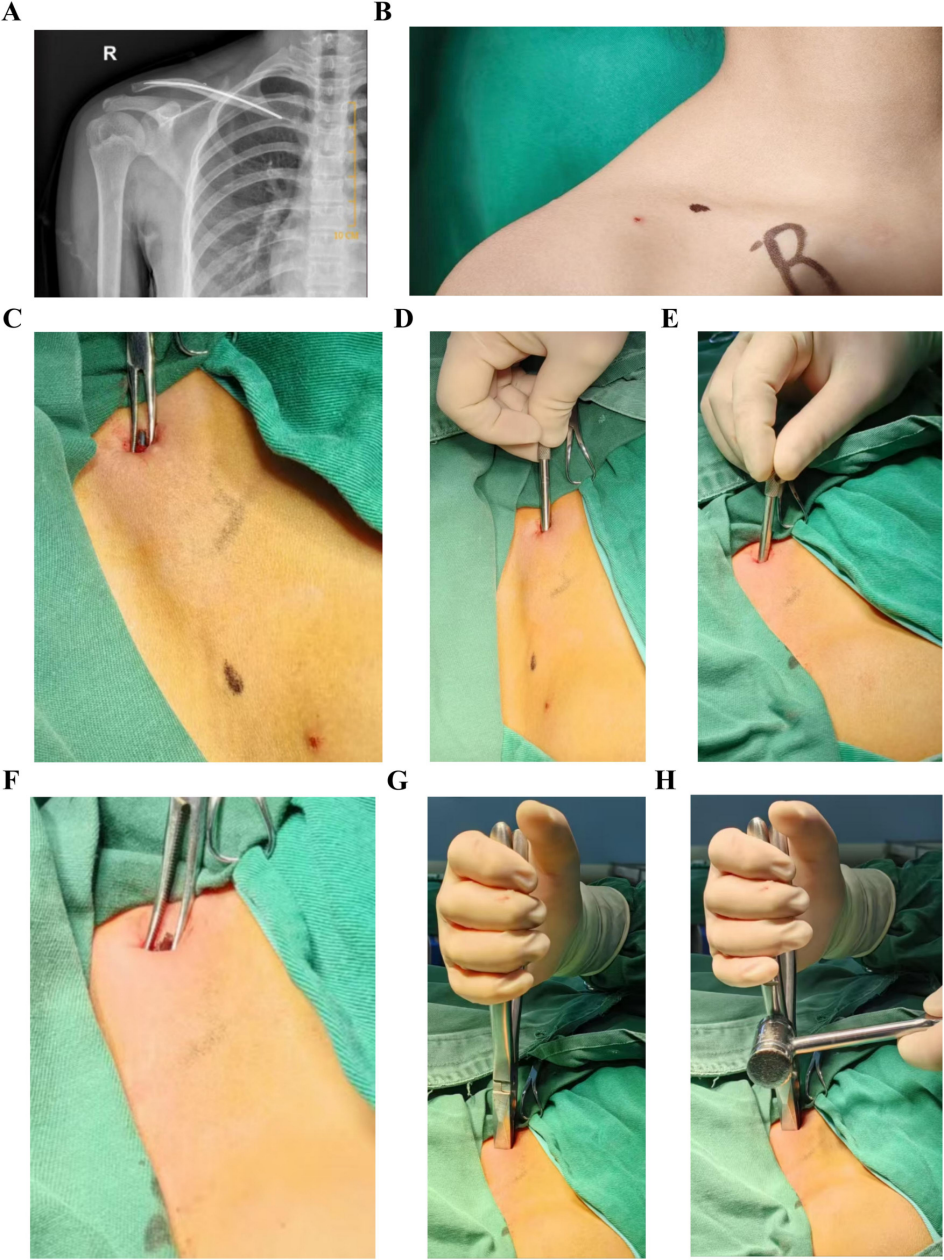
K-wire sleeve-assisted clavicular ESIN removal. **(A–H)** Clinical case illustration. ESIN, elastic intramedullary nails; K-wire, Kirschner Wire.

In the observation group, ESIN removal was performed with K-wire sleeve assistance. All patients underwent incisions matching the original size or slightly smaller, followed by layered dissection to expose the ESIN tip. A K-wire sleeve of corresponding diameter was inserted into the ESIN tip, and the tip was then bent. Subsequently, the clamping instruments could easily grasp the nearly vertically bent ESIN tip, and the ESIN was extracted by hammer strikes applied to the clamp. The wound was irrigated, sutured in layers, and covered with sterile dressings. Postoperative wound dressings were changed regularly (Figures 6–8). We applied this technique for ESIN removal for adult clavicles (Figure 6) and fibulae, as well as for pediatric tibiae (Figure 7), clavicles (Figure 8), ulnae, radii, femora, and humeri.

### 2.3 Observation indicators

We monitored several perioperative indicators by documenting the key surgical parameters for each case, including incision length, operative time, and surgical complications. In addition, we monitored the operative time, defined as the duration from exposure of the tail end of each ESIN (accounting for variability in anatomical removal sites and the number of nails) to the complete removal of all elastic intramedullary nails. We also recorded all complications, including postoperative complications, such as incision infections.

### 2.4 Statistical methods

Data were analyzed with SPSS version 25.0 (IBM Corp., Armonk, NY.). The Shapiro–Wilk test was used to test continuous variables (operative time, incision length) for normality. Normally distributed data (mean ± standard deviation [SD]) were compared by *t*-tests, while non-normally distributed data (median, inter-quartile range [IQR]) were compared by the Mann–Whitney *U* test. Categorical data are given as *n* (%) and were analyzed by the Chi-squared or Fisher's exact test. Two-tailed tests with *p* < 0.05 were considered significant.

## 3 Results

### 3.1 Comparative analysis of preoperative baseline data

Thirty-two patients were retrospectively assigned to two groups based on surgical method: the observation group (*n* = 17) and control group (*n* = 15). The control group comprised 10 males and 5 females, with removal sites including the tibia (2 cases), femur (3 cases), humerus (2 cases), ulna (6 cases), radius (5 cases), and fibula (2 cases); in total, 27 ESINs were removed. The observation group included 12 males and 5 females, with removal sites covering the tibia (5 cases), clavicle (4 cases), femur (3 cases), humerus (2 cases), ulna (2 cases), and radius (3 cases); in total, 30 ESINs were removed. No significant differences were observed between the groups in terms of age, gender, or surgical site distribution (*p* > 0.05) (Table 1).

**Table 1:** Comparative analysis of patient baseline characteristics.

Parameter	Observation group (*n* = 17)	Control group (*n* = 15)	*p*-value
Gender (F/M)	5⁄12	5⁄10	1.000
Surgical site			
Tibia	5 (29.4%)	2 (13.3%)	0.403
Femur	3 (17.6%)	1 (6.7%)	1.000
Humerus	2 (11.8%)	2 (13.3%)	1.000
Ulna	2 (11.8%)	2 (13.3%)	1.000
Radius	3 (17.6%)	1 (6.7%)	0.424
Fibula	0 (0%)	2 (13.3%)	0.212
Clavicle	4 (23.5%)	0 (0%)	0.104
Age (years)	12 (9–13)	11 (9–12)	0.909

### 3.2 Comparative analysis of intraoperative parameters

Intraoperative outcomes for the observation group (17 patients, 30 ESINs) and control group (15 patients, 27 ESINs) are shown in Table 2. The observation group experienced significantly a shorter operative time and incision length compared to the control group (*p* < 0.001).

**Table 2:** Intraoperative parameters (Per nail, mea*n* ± SD).

Parameter	Observation group	Control group	*t*-value	*p*-Value
Operative time (min/nail)	4.65 ± 1.12	11.33 ± 1.47	−14.31	<0.001
Incision length (cm/nail)	0.95 ± 0.11	1.43 ± 0.33	−5.38	<0.001

Operative time was calculated from exposure of the nail tail to complete removal of each ESIN.

### 3.3 Comparative analysis of postoperative complications

No instances of postoperative incision infections, intraoperative nerve injuries, or vascular injuries were observed for any of the 32 patients.

## 4 Discussion

Currently, there are limited reports on the improvement of ESIN removal techniques. However, the increasing emergence of various ESIN removal devices highlights the ongoing potential for methodological refinement in this field, thus highlighting the necessity and clinical value of such research. Traditional removal methods often require extending the original incision to fully expose the ESIN tip, thus facilitating grasping with clamping instruments for extraction. This incision extension compromises the minimally invasive benefits achieved in prior surgical procedures.

**Figure 9:**
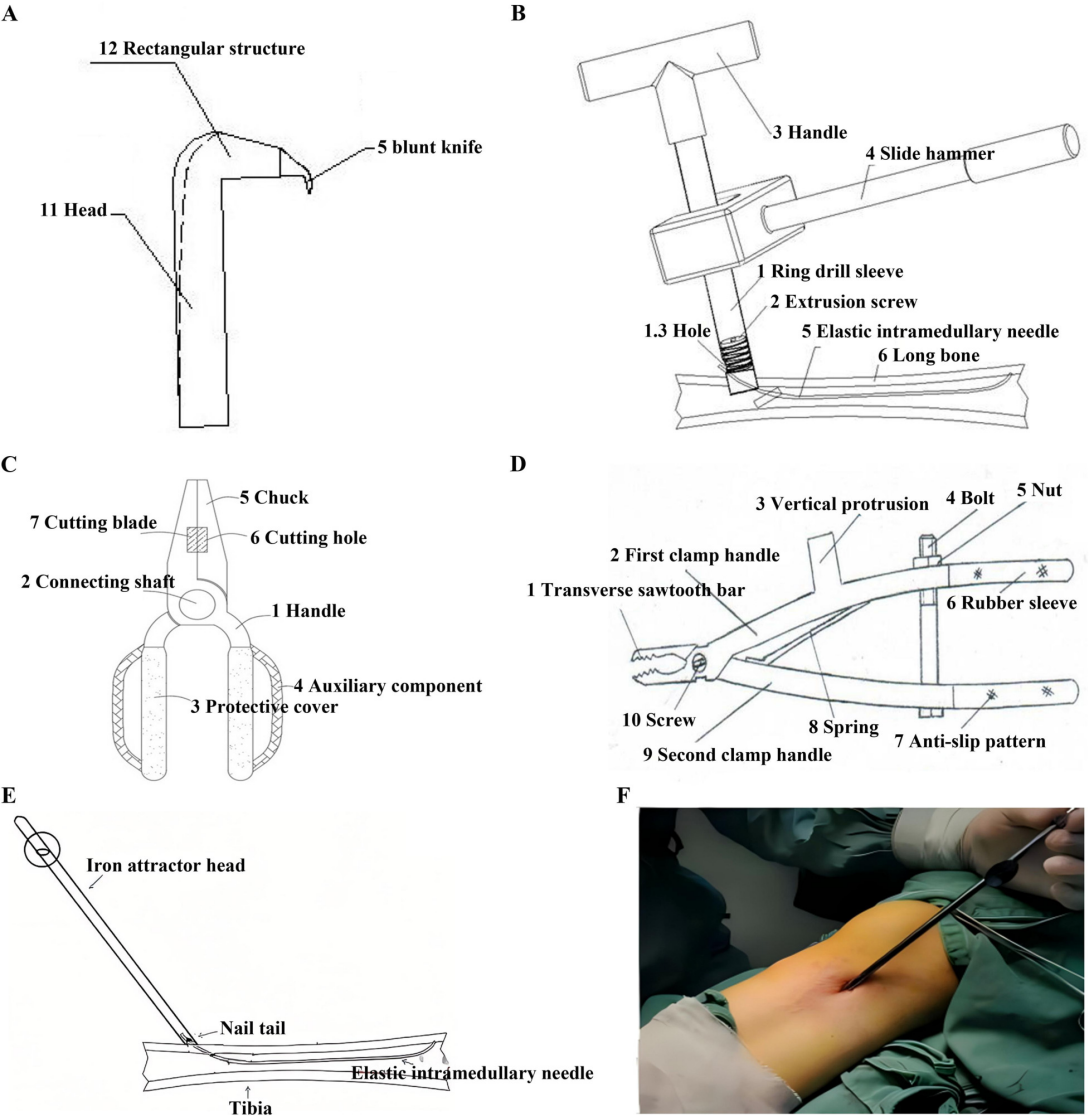
Novel ESIN removal tools developed in China. **(A–D)** Sealed extractor, S-shaped lever, ergonomic system, and multi-component device; **(E,F)** metal suction tip application. ESIN, elastic intramedullary nails.

Current ESIN extraction techniques face numerous challenges (Figures 1–3), such as: instrument limitations, nail tip angulation and the length of the nail tip. To overcome these challenges, researchers have proposed multiple innovative methods. Notable domestic advancements in ESIN extraction instrumentation include: the ESIN Sealing Extractor developed by Ren et al. (4), comprising a cannulated ring, compression screw, and threaded components (Figure 9A); the novel Pediatric ESIN Extractor developed by Huang et al. (5) featuring crossed S-shaped primary and secondary handles generating scissor-type leverage (Figure 9B); the dedicated ESIN Removal System incorporating ergonomic grips, linkage shafts, protective sleeves, and auxiliary modules developed by Cheng et al. (6) (Figure 9C); and a multi-component extractor engineered with primary/secondary clamping handles, screw-spring mechanisms, bolted fasteners, and rubber sheaths developed by Huang et al. (7) (Figure 9D). On an international level, Lascombes osteotome-assisted technique (8) often necessitates larger incisions while involving bone removal with osteotomes. Finally, Gautam et al. (9) reported the application of metal suction tips to facilitate ESIN tip bending during extraction procedures (Figures 9E,F).

**Figure 10:**
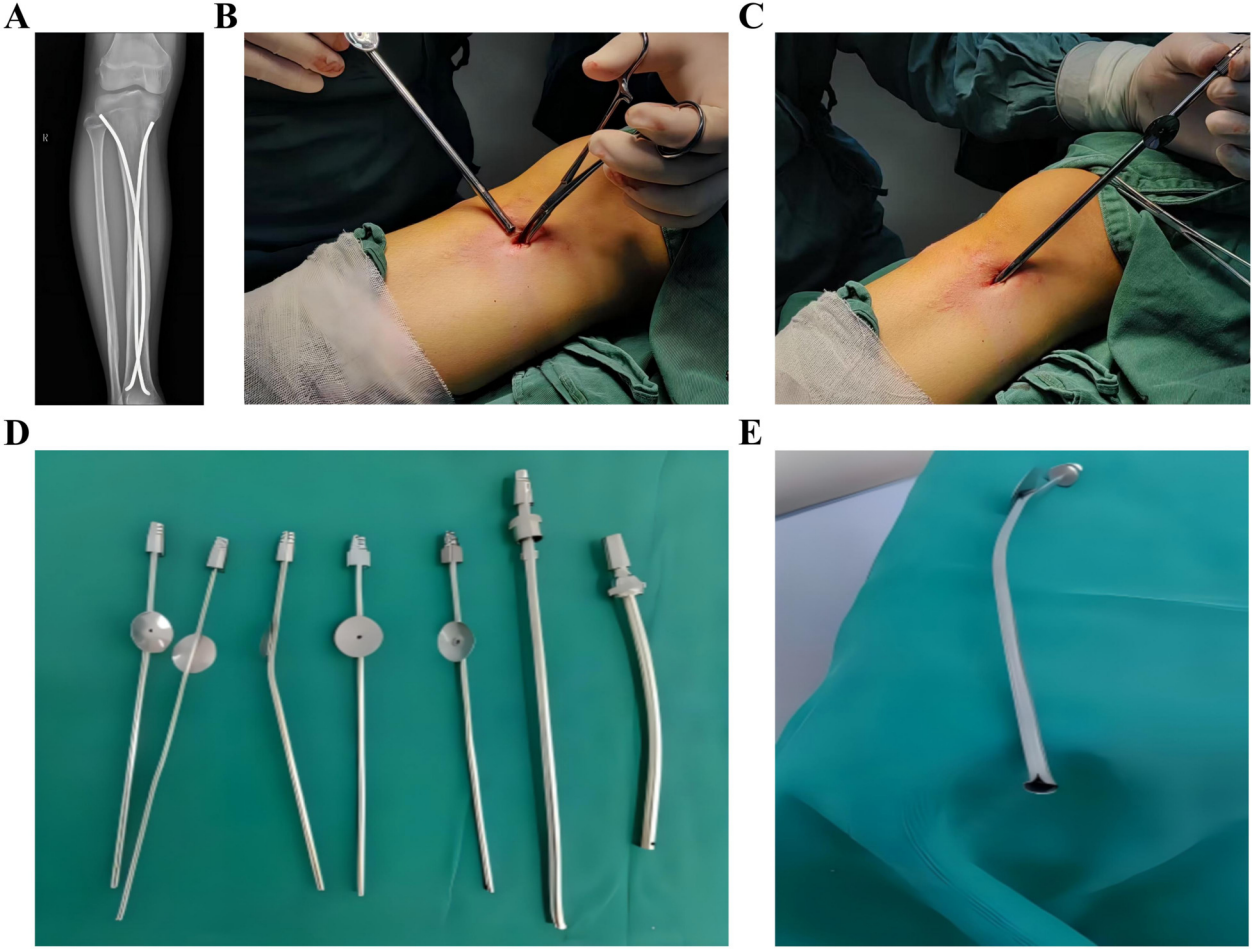
Mechanical failure of metal suction tips. **(A–E)** Deformation and microfractures.

While these methods possess respective advantages, they are not without limitations. For instance, although numerous newly developed instruments have improved the removal of ESIN, these devices are associated with numerous drawbacks such as complex assembly requirements and the necessity for specialized procurement. Metal suction tips are common surgical instruments in operating rooms, offering the advantages of requiring no additional procurement and providing ease of use. However, as their primary function is fluid aspiration during surgical procedures, their resistance to bending forces is inherently limited. In our experimental attempts to bend the tips of ESIN using these devices, all tested models of metal suction tips exhibited significant mechanical damage, including bending deformation and fracture formation (Figures 10A–E), thereby preventing their re-application. Compared to Gautam's metal suction technique, the K-wire sleeve avoids mechanical damage (Figures 10A–E) and offers re-usable instrumentation, thus reducing costs. Additionally, unlike Lascombes osteotome method, our technique preserves bone integrity by eliminating the need for cortical chiseling.

**Figure 11:**
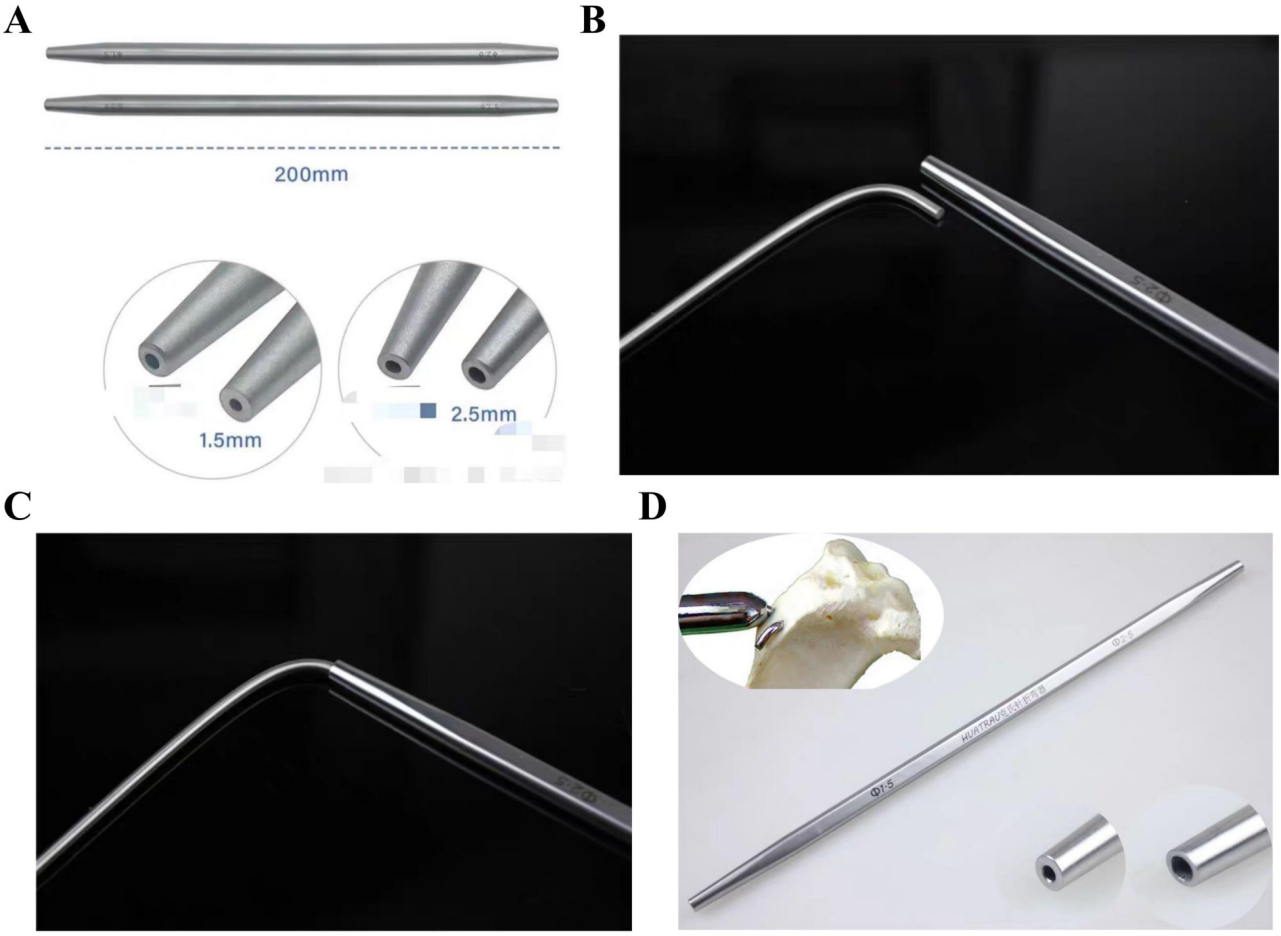
Limitations of K-wire benders. **(A–D)** Diameter incompatibility and insertion challenges. K-wire, Kirschner Wire.

Fundamentally analogous to K-wire, ESINs share similar structural properties, theoretically allowing removal through analogous techniques such as bending the nail tail with K-wire benders, a strategy that could significantly reduce operative time (Figures 11A–D). However, standard K-wire benders are only available in diameters ranging from 0.6 mm to 2.5 mm, resulting in inherent limitations when applied to ESINs exceeding 2.5 mm in diameter.

**Figure 12:**
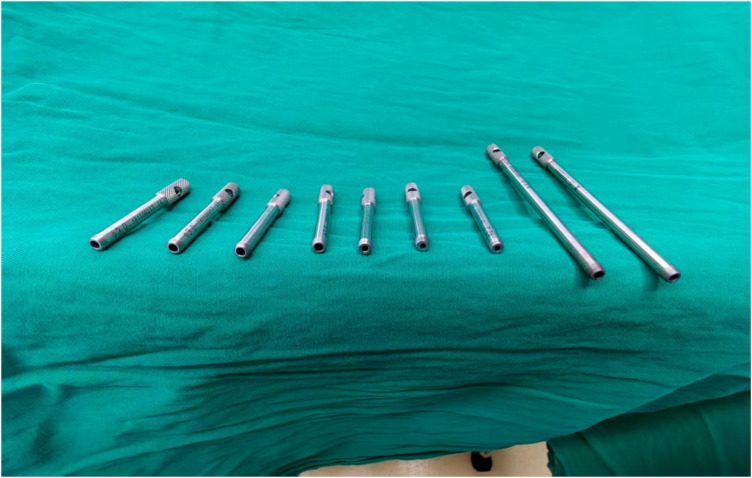
Durability of K-wire sleeves: No structural damage post-procedure. K-wire, Kirschner Wire.

The mechanism of utilizing K-wire sleeves for ESIN removal can be summarized as four steps. First, the primary distinction of this method from traditional methods lies in focusing on bending the ESIN tip; once bent, clamping instruments can securely grip the tip without slippage. Second, multiple diameter specifications can accommodate varying ESIN sizes. Third, the tapered design (thin tip to thickened tail) allows easy insertion into the ESIN tip, thus eliminating the need for full exposure or incision enlargement. Finally, the sleeve resists deformation during bending, with no instances of sleeve damage observed in any of the clinical cases described herein (Figure 12).

This study is a retrospective single-center investigation with a small number of cases. Consequently, it is inevitable that there are inherent limitations in the design of this study, thus necessitating future validation through prospective studies with expanded cohorts. However, our findings suggest that this approach represents a viable treatment option worthy of clinical application.

## 5 Conclusion

In this study, we addressed the status of ESIN removal as this remains a niche and under-recognized field. Our analysis demonstrates that the K-wire sleeve technique provides a reliable novel method for ESIN extraction, effectively improving procedural quality when compared to traditional methods.

## Data Availability

The original contributions presented in the study are included in the article/Supplementary Material, further inquiries can be directed to the corresponding author/s.
