# Supplementary material for: The surgical efficiency of Kirschner wire sleeve-assisted removal of elastic intramedullary nails: a comparative study
Source: Front Pediatr. 2026 Jan 29;13:1689452. doi: 10.3389/fped.2025.1689452 (PMC12894239; doi:10.3389/fped.2025.1689452)

Table 1 Clinical outcomes

Case Region Amount Surgery duration (min) Incision length(cm) Complications Blood loss(ml)

1 Tibia Two 7min R:1.1cm L:0.8cm None 22ml

2 Femur Two 9min R:1.2cm L:1.0cm None 23ml

3 Clavicle One 5min 0.8cm None 10ml

4 Clavicle One 6min 0.8cm None 10ml

5 Humerus Two 10min R:1.0cm L:0.9cm None 24ml

6 Humerus Two 9min R:1.0cm L:1.1cm None 20ml

7 Femur Two 9min R:1.2cm L:1.1cm None 23ml

8 Clavicle One 7min 0.9cm None 11ml

9 Femur Two 9min R:1.2cm L:1.0cm None 21ml

10 Tibia＆Fibula Three 12min Fibula：0.7cm None 28ml

Tibia（R）:0.9cm

Tibia（L）:0.8cm

11 Tibia Two 8min R:1.0cm L:0.9cm None 24ml

12 Tibia Two 8min R:1.0cm L:0.9cm None 20ml

13 Radius One 5min 0.9cm None 10ml

14 Radius＆Ulna Two 7min Radius:0.9cm Ulna:0.7cm None 20ml

15 Tibia Two 8min R:0.8cm L:1.0cm None 22ml

16 Radius＆Ulna Two 7min Radius:1.0cm Ulna:0.8cm None 20ml

17 Clavicle One 5min 0.8cm None 10ml

Table 2 Clinical outcomes

Case Region Amount Surgery duration (min) Incision length(cm) Complications Blood loss(ml)

1 Fibula One 11min 1.0cm None 15ml

2 Tibia Two 25min R:1.5cm L:1.5cm None 24ml

3 Femur Two 20min R:2.0cm L:2.0cm None 25ml

4 Humerus Two 19min R:1.5cm L:1.6cm None 20ml

5 Radius＆Ulna Two 22min Radius:1.4cm Ulna:1.3cm None 20ml

6 Humerus Two 21min R:1.7cm L:1.6cm None 20ml

7 Femur Two 19min R:1.8cm L:2.0cm None 25ml

8 Femur Two 20min 2.0cm None 21ml

9 Tibia Two 23min R:1.5cm L:1.5cm None 20ml

10 Radius＆Ulna Two 27min Radius:1.8cm Ulna:1.3cm None 24ml

11 Radius＆Ulna Two 25min Radius:1.6cm Ulna:1.5cm None 20ml

12 Radius＆Ulna Two 24min Radius:1.5cm Ulna:1.5cm None 20ml

13 Radius＆Ulna Two 23min Radius:1.5cm Ulna:1.4cm None 20ml

14 Fibula One 12min 1.0cm None 15ml

15 Radius One 14min 1.5cm None 15ml

**Surgical time: Considering that the locations for removing elastic intramedullary nails vary and the number of elastic intramedullary nails at each location also varies, surgical time starts from the exposure of the tail of each elastic intramedullary nail and ends when the elastic intramedullary nail is removed.**

Based on the raw data of Table 1 and Table 2, calculate the normalized parameters case by case and revise the mean values as follows:

### Normalization Method

1. Surgical time per root (min) = Surgical time ÷ Amount
2. Incision length per root (cm) = Total incision length (including multiple sites) ÷ Amount

### ****Table 1**** Case-by-case normalized calculation

| **Case** | Surgical time per root（min） | Incision length per root（cm） | Blood loss per root（ml） |
| --- | --- | --- | --- |
| 1 | 7 ÷ 2 = **3.50** | (1.1+0.8)/2 = **0.95** | 22 ÷ 2 = **11.00** |
| 2 | 9 ÷ 2 = **4.50** | (1.2+1.0)/2 = **1.10** | 23 ÷ 2 = **11.50** |
| 3 | 5 ÷ 1 = **5.00** | 0.8 ÷ 1 = **0.80** | 10 ÷ 1 = **10.00** |
| 4 | 6 ÷ 1 = **6.00** | 0.8 ÷ 1 = **0.80** | 10 ÷ 1 = **10.00** |
| 5 | 10 ÷ 2 = **5.00** | (1.0+0.9)/2 = **0.95** | 24 ÷ 2 = **12.00** |
| 6 | 9 ÷ 2 = **4.50** | (1.0+1.1)/2 = **1.05** | 20 ÷ 2 = **10.00** |
| 7 | 9 ÷ 2 = **4.50** | (1.2+1.1)/2 = **1.15** | 23 ÷ 2 = **11.50** |
| 8 | 7 ÷ 1 = **7.00** | 0.9 ÷ 1 = **0.90** | 11 ÷ 1 = **11.00** |
| 9 | 9 ÷ 2 = **4.50** | (1.2+1.0)/2 = **1.10** | 21 ÷ 2 = **10.50** |
| 10 | 12 ÷ 3 = **4.00** | (0.7+0.9+0.8)/3 = **0.80** | 28 ÷ 3 = **9.33** |
| 11 | 8 ÷ 2 = **4.00** | (1.0+0.9)/2 = **0.95** | 24 ÷ 2 = **12.00** |
| 12 | 8 ÷ 2 = **4.00** | (1.0+0.9)/2 = **0.95** | 20 ÷ 2 = **10.00** |
| 13 | 5 ÷ 1 = **5.00** | 0.9 ÷ 1 = **0.90** | 10 ÷ 1 = **10.00** |
| 14 | 7 ÷ 2 = **3.50** | (0.9+0.7)/2 = **0.80** | 20 ÷ 2 = **10.00** |
| 15 | 8 ÷ 2 = **4.00** | (0.8+1.0)/2 = **0.90** | 22 ÷ 2 = **11.00** |
| 16 | 7 ÷ 2 = **3.50** | (1.0+0.8)/2 = **0.90** | 20 ÷ 2 = **10.00** |
| 17 | 5 ÷ 1 = **5.00** | 0.8 ÷ 1 = **0.80** | 10 ÷ 1 = **10.00** |

### ****Table 2**** Case-by-case normalized calculation

| **Case** | Surgical time per root（min） | Incision length per root（cm） | Blood loss per root（ml） |
| --- | --- | --- | --- |
| 1 | 11 ÷ 1 = **11.00** | 1.0 ÷ 1 = **1.00** | 15 ÷ 1 = **15.00** |
| 2 | 25 ÷ 2 = **12.50** | (1.5+1.5)/2 = **1.50** | 24 ÷ 2 = **12.00** |
| 3 | 20 ÷ 2 = **10.00** | (2.0+2.0)/2 = **2.00** | 25 ÷ 2 = **12.50** |
| 4 | 19 ÷ 2 = **9.50** | (1.5+1.6)/2 = **1.55** | 20 ÷ 2 = **10.00** |
| 5 | 22 ÷ 2 = **11.00** | (1.4+1.3)/2 = **1.35** | 20 ÷ 2 = **10.00** |
| 6 | 21 ÷ 2 = **10.50** | (1.7+1.6)/2 = **1.65** | 20 ÷ 2 = **10.00** |
| 7 | 19 ÷ 2 = **9.50** | (1.8+2.0)/2 = **1.90** | 25 ÷ 2 = **12.50** |
| 8 | 20 ÷ 2 = **10.00** | 2.0 ÷ 2 = **1.00** | 21 ÷ 2 = **10.50** |
| 9 | 23 ÷ 2 = **11.50** | (1.5+1.5)/2 = **1.50** | 20 ÷ 2 = **10.00** |
| 10 | 27 ÷ 2 = **13.50** | (1.8+1.3)/2 = **1.55** | 24 ÷ 2 = **12.00** |
| 11 | 25 ÷ 2 = **12.50** | (1.6+1.5)/2 = **1.55** | 20 ÷ 2 = **10.00** |
| 12 | 24 ÷ 2 = **12.00** | (1.5+1.5)/2 = **1.50** | 20 ÷ 2 = **10.00** |
| 13 | 23 ÷ 2 = **11.50** | (1.5+1.4)/2 = **1.45** | 20 ÷ 2 = **10.00** |
| 14 | 12 ÷ 1 = **12.00** | 1.0 ÷ 1 = **1.00** | 15 ÷ 1 = **15.00** |
| 15 | 14 ÷ 1 = **14.00** | 1.5 ÷ 1 = **1.50** | 15 ÷ 1 = **15.00** |


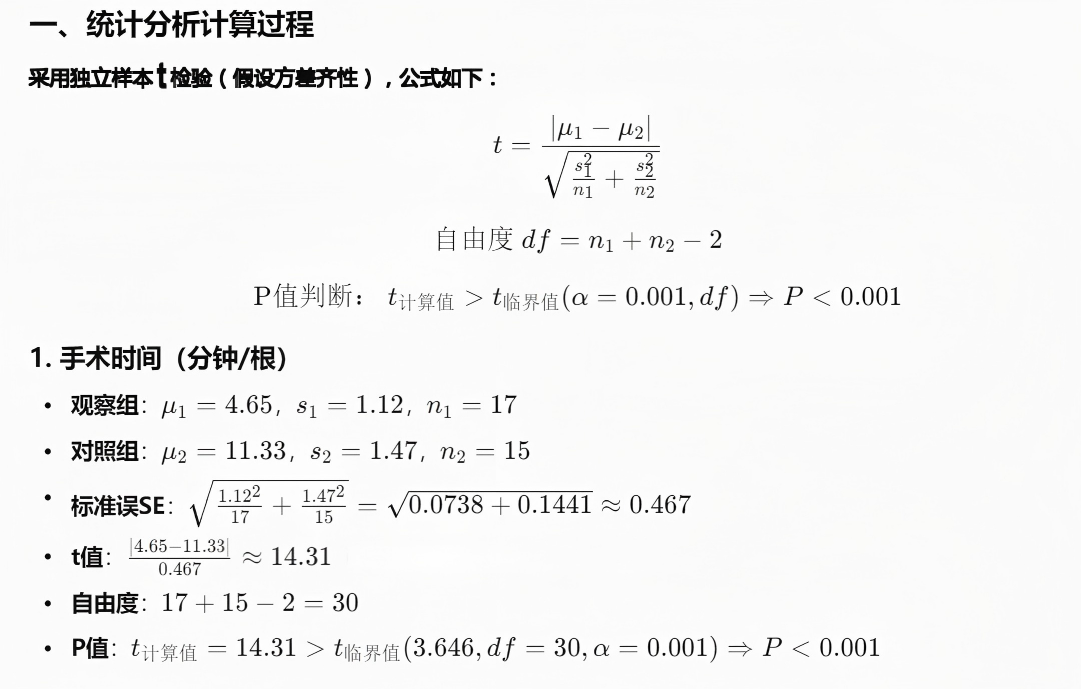

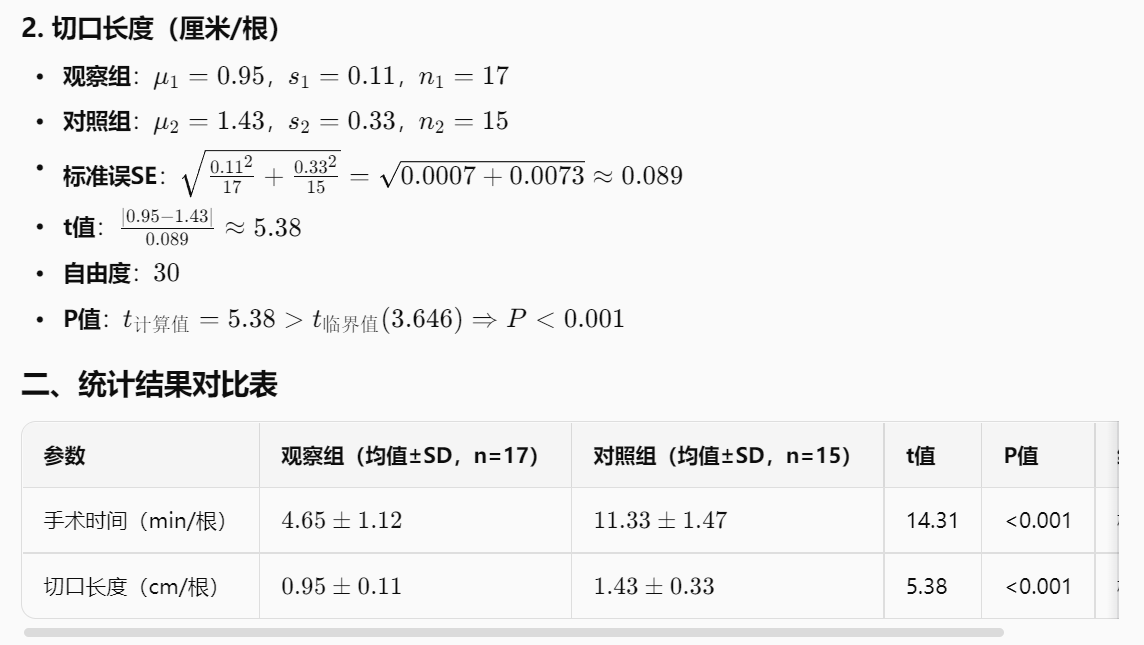


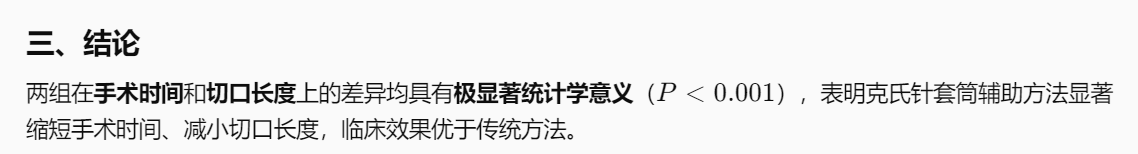

Supplement: Supplementary file 1 [file Table1.docx]
